# Src-dependent EGFR transactivation regulates lung inflammation via downstream signaling involving ERK1/2, PI3Kδ/Akt and NFκB induction in a murine asthma model

**DOI:** 10.1038/s41598-017-09349-0

**Published:** 2017-08-30

**Authors:** Ahmed Z. El-Hashim, Maitham A. Khajah, Waleed M. Renno, Rhema S. Babyson, Mohib Uddin, Ibrahim F. Benter, Charles Ezeamuzie, Saghir Akhtar

**Affiliations:** 1 Department of Pharmacology & Therapeutics, Faculty of Pharmacy, Kuwait City, Kuwait; 2 Department of Anatomy, Faculty of Medicine, Kuwait University, Kuwait City, Kuwait; 3 Respiratory, Inflammation & Autoimmunity iMED, AstraZeneca R&D Gothenburg, Mölndal, Sweden; 4 Department of Pharmacology & Toxicology, Faculty of Medicine, Kuwait University, Kuwait City, Kuwait; 5 Faculty of Medicine, Eastern Mediterranean University, Famagusta, Cyprus

## Abstract

The molecular mechanisms underlying asthma pathogenesis are poorly characterized. In this study, we investigated (1) whether Src mediates epidermal growth factor receptor (EGFR) transactivation; (2) if ERK1/2, PI3Kδ/Akt and NF-κB are signaling effectors downstream of Src/EGFR activation; and (3) if upstream inhibition of Src/EGFR is more effective in downregulating the allergic inflammation than selective inhibition of downstream signaling pathways. Allergic inflammation resulted in increased phosphorylation of EGFR, Akt, ERK1/2 and IκB in the lung tissues from ovalbumin (OVA)-challenged BALB/c mice. Treatment with inhibitors of Src (SU6656) or EGFR (AG1478) reduced EGFR phosphorylation and downstream signaling which resulted in the inhibition of the OVA-induced inflammatory cell influx in bronchoalveolar lavage fluid (BALF), perivascular and peribronchial inflammation, fibrosis, goblet cell hyper/metaplasia and airway hyper-responsiveness. Treatment with pathway-selective inhibitors for ERK1/2 (PD89059) and PI3Kδ/Akt (IC-87114) respectively, or an inhibitor of NF-κB (BAY11-7085) also reduced the OVA-induced asthmatic phenotype but to a lesser extent compared to Src/EGFR inhibition. Thus, Src via EGFR transactivation and subsequent downstream activation of multiple pathways regulates the allergic airway inflammatory response. Furthermore, a broader upstream inhibition of Src/EGFR offers an attractive therapeutic alternative in the treatment of asthma relative to selectively targeting the individual downstream signaling effectors.

## Introduction

Chronic airways inflammation resulting in airway structural remodeling and the functional changes such as airway obstruction and airway hyperresponsivessness (AHR) are pathological hallmarks of asthma^1^. Airway epithelial cells (AEC) are increasingly being recognized as important players in the pathogenesis of asthma and are appropriately positioned at the interface between the host mucosal surface and environmental insults^2^. They secrete many bioactive mediators that regulate key inflammatory responses, such as chemotaxis, cell activation, apoptosis and airway remodeling^2^. Epidermal growth factor (EGF) is an important epithelial-derived mediator that signals through EGF receptor (EGFR) and has been implicated in numerous disease such as cancer, cardiovascular disease, chronic renal disease, diabetes and allergic diseases such as asthma^3–10^.

Accumulating evidence indicates that EGFR-dependent signaling contributes to asthma pathophysiology^11^. For example, asthmatic airways show increased EGF and EGFR immunoreactivity in the bronchial epithelium, airway glands, smooth muscle and basement membrane and this correlates with subepithelial basement membrane thickening^3^. Preclinical animal models of asthma have further shown that inhibition of EGFR activation reduces allergen-induced eosinophil influx, MUC51 protein expression in bronchoalveolar lavage (BAL), AHR and epithelial and airway smooth muscle (ASM) remodeling^5, 12, 13^. Of relevance, EGF can induce the airway epithelium, from more severe asthmatics, to generate pro-neutrophilic factors that can have profound chemotactic and apoptosis-delaying actions *ex vivo* ^14^.

Whilst EGFR is typically activated by cognate ligands such as EGF heparin-binding (HB)-EGF or amphiregulin, it can also be transactivated by other mechanisms such as Src kinases. Members of the tyrosine Src family of kinase (SKF) via cytokine and growth factor-dependent signaling are implicated in regulating asthmatic responses^15–18^ and network-biology modeling has identified this kinase family as a potential druggable target for asthma^19^. Recently, we have also shown that hyperglycemia results in increased EGFR transactivation that is SFK dependent^20^. These studies show an important role for SFK in upstream EGFR signaling. However, whether SFK mediate EGFR transactivation in the context of asthmatic responses is unknown.

Multiple signaling pathways downstream of EGFR, including the classical cytosolic ras/raf/ ERK1/2, p38 mitogen-activated protein kinase (MAPK), PI3K/Akt/mTOR, IκB-α, ROCK and eNOS, have been reported to be involved in mediating a variety of asthmatic responses^21–23^. For example, ERK1 has also been shown to play an important role in Th2 cell differentiation and development of experimental asthma models^24^. Class I PI3K isoforms can play distinct signaling roles in a variety of asthmatic responses, including neutrophilic inflammation^14, 25^. Also, blockade of PI3Kδ or the transcriptional factor NFκB inhibits the manifestation of the asthma phenotype such as serum IgE, OVA-specific IgE, tissue eosinophilia and mucus production in murine models of asthma^22, 26^. Whilst the importance of these signaling pathways is recognized in asthma, what remain unclear is whether they are downstream of EGFR and/or Src and their relative contribution in regulating the asthma pathobiology. The objective of this study was to (1) to determine if SFK mediates EGFR transactivation; (2) identify if ERK1/2, PI3Kδ/Akt and NFκB are downstream signaling effectors of EGFR in a murine model of allergic airway inflammation *in vivo* and; (3) to assess whether upstream SFK/EGFR inhibition is more effective than selective inhibition of downstream effectors.

## Methods

### Animals

Male BALB/c mice (6–8 weeks old) used in this study were maintained under temperature-controlled conditions with an artificial 12 h light/dark cycle and were allowed standard chow and water *ad libitum*. All studies involving animals are reported in accordance with the principles of NC3Rs’ ARRIVE guidelines for reporting humane animal research. All experimental protocols were approved by the “Health Science Center Animal Welfare Committee” and complied with regulations for the animal care and ethical use of Laboratory Animals in the Health Sciences Center, Kuwait University.

### Immunization and intranasal challenge and drug treatment protocols

BALB/c mice were immunized once by *i.p*. injection of 10 µg ovalbumin (OVA) in 0.2 ml of alu-Gel-S on day 0. Ten days later, mice were intranasally (*i.n*.) challenged with OVA (30 µg in 50 µL PBS) or PBS, once daily, over four consecutive days.

To investigate the importance of EGFR transactivation in allergic airway inflammatory responses, six treatment groups (A–F, 11–18 animals per group) were established. Mice in groups A and B were pretreated *i.p*. with 0.2 ml of the vehicle for AG1478, 1 h before each intranasal challenge with PBS and OVA, respectively. In the same manner, groups C, D and E were pretreated with the same volume of AG1478 at 0.03, 0.06 and 0.1 mg/kg, respectively, and group F was pretreated with dexamethasone (1 mg/kg), 1 h before each *i.n*. challenge with OVA.

To determine whether EGFR transactivation is dependent on Src kinase activation, six treatment groups (A–F, 10–26 animals per group) were established. Mice in groups A and B were pretreated intranasally with 0.2 ml of the vehicle for SU6656, 1 h before each *i.n*. challenge with PBS and OVA, respectively. In the same manner, groups C, D and E were pretreated with the same volume of SU6656 at 1, 4 and 8 mg/kg, respectively, and group F with dexamethasone (1 mg/kg), 1 h before each *i.n*. challenge with OVA.

Similarly, to investigate if ERK1/2, PI3Kδ and NFκB are signaling effectors downstream of EGFR transactivation, six treatment groups (A–F, 10–30 animals per group) were established. Mice in groups A and B were pretreated intranasally with 0.2 ml of the vehicle for the drugs.﻿ Groups C, D and E were pretreated with the same volume of three different drugs (PD 98059, IC-87114 and BAY 11-7085, respectively) at 10 mg/kg, 10 mg/kg and 0.3 mg/kg respectively, and group F with dexamethasone (1 mg/kg), 1 h before each *i.n*. challenge with OVA. These doses were chosen from previous studies where they were shown to be effective^22, 26, 27^. In all of the study groups performed, dexamethasone (1 mg/kg) was used as a positive control to compare the effects of AG1478, SU6656, PD 89059, IC-87114 and BAY 11-7085 against. Glucocorticoids are the mainstay therapy in asthma and we have also shown in the same experimental model that dexamethasone, (1 mg/kg), effectively inhibits the OVA- mediated allergic airway inflammatory responses^26, 21^.

For all the three treatment protocol, two set of experiments were conducted 24 h after the last *i.n*. challenge: one set used for the bronchoalveolar lavage (BAL)/cytology/histology/immununohistochemistry(IHC)/immunofluorescence/(IF) studies whereas the other set was used for the AHR assessment. For the histology/IHC/IF studies, we mainly assessed the dose that had the highest effect based on the cytology data.

### BAL Fluid Cell Counts and Lung Histology

BAL fluid was collected and cells enumerated using standard morphologic criteria as described previously^21^.

Lung specimens were prepared for histology and immunohistology as previously described^21^. Lung sections were also stained by H&E stain, Masson’s Trichrome stains and periodic acid–Schiff (PAS) and blindly scored using the scoring system (1 = normal, 2 = mild, 3 = moderate, 4 = severe, and 5 = highly severe) as described previously^21^.

### Immunohistochemistry

The tissue sections were deparaffinized in xylene and rehydrated in graded series of alcohol. Tissues were treated for 15 min in 3% H_2_O_2_ followed by 30 min in 50 mM glycine + 0.1% sodium borohydride in distilled water, washed in PBS and blocked with 2% normal goat serum (NGS) before applying the rabbit polyclonal primary antibody (EGFR and phospho- EGFR, Santa Cruz) overnight. The slides were washed and treated with biotinylated goat-anti rabbit IgG (1:200) and 1% NGS for 1 h at room temperature. Slides were then washed three times with PBS and treated with avidin biotin complex with 0.1% tween 20 for 1 h at room temperature. Sections were colour developed with 3-diaminobenzidine as a chromogen (brown) and counter stained with haematoxylin (blue). The slides were dehydrated by passing through graded series of alcohol, cleared in xylene before mounting a coverslip using DPX Mountant.

### Immunofluorescence

Tissues were processed as described above. Immunofluorescence studies were done according to a recently published study^28^. In brief, lung sections were then incubated in blocking solution (5% bovine serum albumin (BSA) + 0.3% Triton X-100 in PBS) for 1 h, followed by incubation overnight at 4 °C with primary antibodies [pEGFR, pERK1/2, pAkt, pI-κB, (1:50 - 1:100 dilution); Cell Signaling, USA], diluted in 1% blocking solution. Subsequently, sections were washed and incubated with secondary antibody conjugated to Alexa Fluor 555 (Goat anti rabbit SFX kit; Life Technologies, USA, 1:400 dilution) for 2 h at room temperature in the dark. pI-κB was measured as a surrogate marker for NF-κB activation. After washes in PBS sections were stained with 4’,6 diamidino-2- phenylindole and mounted. Images were captured on a ZEISS LSM 700 confocal microscope and fluorescence intensity estimated in defined fields using Image J software package.

### Estimation of EGFR expression by Taqman PCR

The right lungs were excised, washed with PBS at 4 °C, snap-frozen in liquid nitrogen and stored at −80 °C. Total RNA was extracted using Trizol reagent (Invitrogen, Carlsbad, CA, USA) according to the manufacturer’s instructions. Two µg of purified RNA (in 20 µl) were converted to cDNA using a high capacity reverse transcriptase kit (Applied Biosystems, CA, USA) and 1 µl cDNA used to amplify EGFR and actin targets using a standard multiplexed TaqMan PCR kit protocol [manufacturer proprietary primer/probe mixes labelled with FAM and VIC]. The 20 µl reactions were performed in a 96-well plate on an Applied Biosystems Fast 7500HT thermocycler by incubation at 95 °C for 10 min, followed by 40 cycles of 95 °C for 15 s and 60 °C for 1 min with a final extension step of 10 min. The raw threshold cycle (CT) values were used to determine target/normaliser ratios by the ΔΔCt method using the spreadsheet developed by Pfaffl^29^.

### Measurement of airway responsiveness

For the measurement of airway responsiveness, airflow was recorded in individual mice using a Buxco FinePointe series RC site (DSI, Wilmington, NC), according to the manufacturer’s guidelines. Briefly, mice were anesthetized with an *i.p*. injection of ketamine/xylazine (1:0.1 mg/kg) cocktail and tracheotomized with a steel 18-gauge cannula. Mice were subsequently mechanically ventilated at a rate of 150 breaths/min, and tidal volume of 0.15 ml, using a computerized small animal ventilator (FinePointe site), as previously described^30, 31^. After 5 min of stabilization, followed by administration of PBS, airway resistance was measured by exposing the mice to aerosolized methalcholine (6.25–50.0 mg/ml, 5 μl per delivery) delivered by an aerogen nebulizer administration, and reported as total lung resistance (*R*_L_) (centimeters H_2_O per ml/sec).

### Isolation of mouse mononuclear cells

Spleen derived mononuclear cells were isolated using the Ficoll-Paque density gradient media^32^. Briefly, mice were euthanized and the spleen was removed, cut into small pieces, and filtered through a 100 µm cell strainer (BD falcon, USA). The filtrate was washed with PBS (1300 rpm, 6 min at 4 °C) and the pellet re-suspended in 2 ml RPMI media, then distributed 3 ml of Ficoll-Paque density gradient media under the cell suspension, and centrifuged (2000 rpm for 30 min at 4 °C). Mononuclear cells were isolated from the high density solution and washed with RPMI media containing 10% fetal bovine serum (FBS) at 1300 rpm for 6 min at 4 °C. The cells were then re-suspended in RPMI media containing 10% FBS at a concentration of 1 × 10^7^cells/ml and viability was >95% as determined by the Trypan blue exclusion test.

### Isolation of murine bone-marrow derived neutrophils

Neutrophils were isolated from murine tibial and femoral bone marrow as described previously^32, 33^. Briefly, mice were euthanized and the femurs and tibias dissected from the animal and the ends of bones removed. The marrow was flushed from the bone with ice-cold 50 ml PBS and then centrifuged at 1300 rpm for 6 min at 4 °C. After harvesting of bone-marrow-derived cells by flushing with PBS, the cells were re-suspended in 3 ml of 52% Percoll and layered on a 3-step Percoll gradient (72%, 64%, and 52% plus cells), and centrifuged (2600 rpm for 30 min at 4 °C). Purified neutrophils were removed from the layer between the 64% and 72% Percoll and washed once with ice-cold PBS and suspended in RPMI culture media containing 20% FBS at a concentration of 10^7^cells/ml. Neutrophil viability was >95% based on Trypan blue exclusion test.

### Assessment of neutrophil chemotaxis (under-agarose assay) *in vitro*

The under agarose chemotaxis assay^32^ was used to determine the effect of BALF on cell chemotaxis. Tissue culture dishes were filled with 3 ml of 0.5% agarose solution. After solidification, three wells (3.5 mm diameter) were created in the gel 2.5 mm apart in a horizontal line. The centre well was loaded with 10 µl of BALF or PBS for control, and the outer wells with 10 µl neutrophils (10^7^ cells/ml) and incubated for 4 h (at 37 °C, 5% CO_2_). Results were analyzed by visual microscopic examination (×100). The degree of chemotaxis was determined by counting the number of cells which migrate towards the source of chemoattractant minus the number migrating away from it.

### Apoptosis assay

Neutrophil apoptosis was assessed using the Annexin V staining method. Freshly isolated murine bone-marrow derived neutrophils or spleen derived mononuclear cells were re-suspended in RPMI + 10% FBS culture media and plated in 35 mm × 10 mm Falcon culture dishes in a density of 1 × 10^6^ cells/ml. Apoptosis was assessed by flow cytometry.

Cells were incubated overnight (at 37 °C/ 5% CO_2_) with vehicle or 100 µl BALF. The cells were then washed twice with ice-cold PBS and once with 1x Annexin-V binding buffer [10x binding buffer; 0.1 M HEPES/NaOH (pH 7.4), 1.4 M NaCl, 25 mM CaCl_2_]. Cell pellets were re-suspended in Annexin-V binding buffer stained for FACS analysis using the PE Annexin V apoptosis detection kit I from BD pharmingen. Cells isolated from each animal were stained in the following manner: cells only (negative control), 1 µl of Annexin V-PE, 1 µl of 7AAD, and 1 µl of Annexin V-PE plus 1 µl 7AAD. All incubations were performed at room temperature for 15 min in the dark.

### Isolation of human blood eosinophils

Fresh blood was obtained from healthy individuals, after getting their informed consent, with no history of allergic disease nor had taken any medication in the last 72 h. The methods and protocol for these experiments were performed in accordance to and approved by the “Ethical Committee of the Faculty of Medicine, Kuwait University”. Granulocytes were isolated from heparinized (10 IU/ml) blood by erythrocyte sedimentation, followed by percoll gradient centrifugation as previously described^34^. Eosinophils were separated using negative selection with the immunomagnetic method as previously described^35^. The eosinophil purity was assessed by differential count of a Wright-Giemsa stained cytosmear and was routinely >98%. Viability was determined by Trypan blue exclusion and exceeded 98%.

### Boyden chamber assay for eosinophil chemotaxis

Peripheral blood derived eosinophils were used for chemotaxis assay using the Boyden chamber as previously described^36^. Purified eosinophils (200 × 10^5^) were placed in the upper wells and in the lower wells, 500 µl of BALF derived from mice challenged with PBS (vehicle) or OVA pretreated with either vehicle, AG1478 (0.1 mg/kg) or dexamethasone (1 mg/kg) and allowed to migrate for 1 h (37 °C/5%CO_2_). The transmigrated cells were determined by counting under the microscope by using a hemocytometer.

### Statistical analysis

All numerical values were expressed as means + S.E.M. Total cell counts represent the number of BALF cells/ml. Differential cell counts represent both the total and an absolute number of each cell type/ml of BALF. Absolute R_L_ values were calculated and used as an index of the airway responsiveness to methacholine. For the histopathology, a semi-quantitative 5-level lung pathology score was used to grade the extent of abnormalities in each microscopic field at 200X. For the airway responsiveness, a two-way repeated measure analysis of variance followed by a Bonferroni post hoc test was used. A one-way analysis of variance (ANOVA) test followed by Newman-Keuls post hoc test was used to compare mean differences between individual groups for the total and differential cell count and histopathological data. For the immunofluorescence data and cell chemotaxis, a ANOVA test followed by Bonferroni post hoc test was used. The mean difference was considered as significant at a probability level of less than 0.05. All results analysis was performed using GraphPad Prism.

## Results

### Effect of AG1478 on EGFR expression and phosphorylation in the OVA- induced asthma phenotype

**Figure 1:**
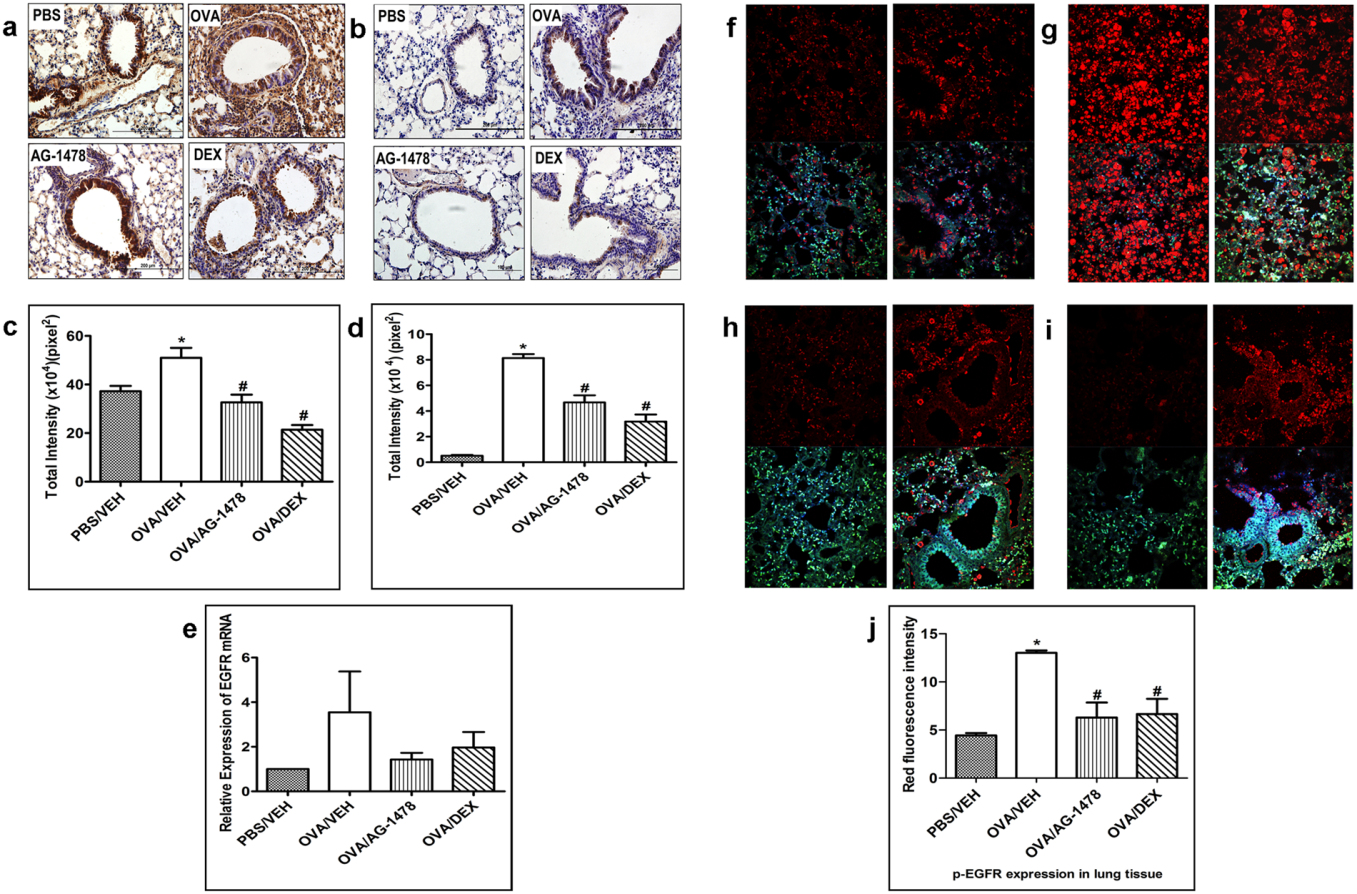
Effect of AG-1478 on immunohistochemistry, RT PCR and immunofluorescence total EGFR immunohistochemical staining **(a)** and phosphorylated EGFR immunohistochemical staining **(b)** distribution photomicrographs of sections of the lung from PBS-challenged and vehicle treated (PBS) (i.p. treated; *n* = 6), OVA-challenged and vehicle treated (OVA) (*i.p*. treated; *n* = 6), OVA-challenged and AG-1478 treated (AG-1478) (0.1 mg/kg; *i.p*. treated; *n* = 6), OVA-challenged and dexamethasone treated (DEX) (1 mg/kg; *i.p*. treated; *n* = 6) groups. Analysis of the total EGFR **(c)** and phosphorylated EGFR **(d)** immunoreactive area in the lung sections of the different treatment groups. Real-time PCR analysis of EGFR expression (*n* = 4) **(e)**. Data are expressed as mean ± SEM. *P < 0.05 *versus* time-matched PBS-challenged mice. ^#^ *P* < 0.05 *versus* time-matched OVA-challenged mice. Immunofluorescent detection of phosphorylated EGFR in lung sections **(f**–**j)**. Lung sections were taken from different treatment groups [(**f)**-PBS/Veh; **(g)**-OVA/Veh, **(h)**-OVA/AG-1478 (0.1 mg/kg) and **(i)**-OVA/Dex (1 mg/kg)] and were immunostained against phosphorylated EGFR. Immunofluorescent (Alexa Fluor) signals are shown on the left side of panels are overlaid with DAPI stain on the right side to show tissue architecture for the conditions indicated. Quantitative assessment of fluorescence intensity of phospho EGFR **(j)** (arbitrary units). Data are expressed as mean ± SEM (*n* = 3). *P < 0.05 *versus* time-matched PBS-challenged mice. ^#^ *P* < 0.05 *versus* time-matched OVA-challenged mice.

To confirm the role of EGFR in our murine model of asthma, we firstly determined the expression and phosphorylation level of EGFR protein by immunohistochemistry (Fig. 1a–d), immunofluorescence (Fig. 1f–j) and EGFR mRNA by RT-PCR (Fig. 1e) in lung tissue from different treatment groups challenged with either OVA or PBS. Intranasal challenge with OVA resulted in a significant (P < 0.05) increase in the total EGFR protein expression compared to the PBS control group as evidenced by IHC (Fig. 1a and c) that was prominent on airway mucosal surface (Fig. 1a). This was associated with an increase in the levels of EGFR mRNA by RT-PCR (Fig. 1e). Furthermore, a significant (*P* < 0.05) increase in pEGFR (up to 16-fold) was observed in the OVA group using immunohistochemistry (Fig. 1b and d) and immunofluorescence (Fig. 1g and j) compared to the PBS control group (Fig. 1b,d,f and j).

Also, treatment with AG1478 (0.1 mg/kg) attenuated the OVA-induced increase in the total EGFR protein (Fig. 1a and c) (*P* < 0.05) and EGFR mRNA (Fig. 1e). AG1478 had a greater inhibitory effect on EGFR phosphorylation (Fig. 1b,d,h and j) than on protein expression (Fig. 1a and c) and was similar to the dexamethasone treatment group (Fig. 1b,d,i and j).

### Effect of AG1478 on OVA- induced inflammatory cell influx, airway remodeling and AHR

**Figure 2:**
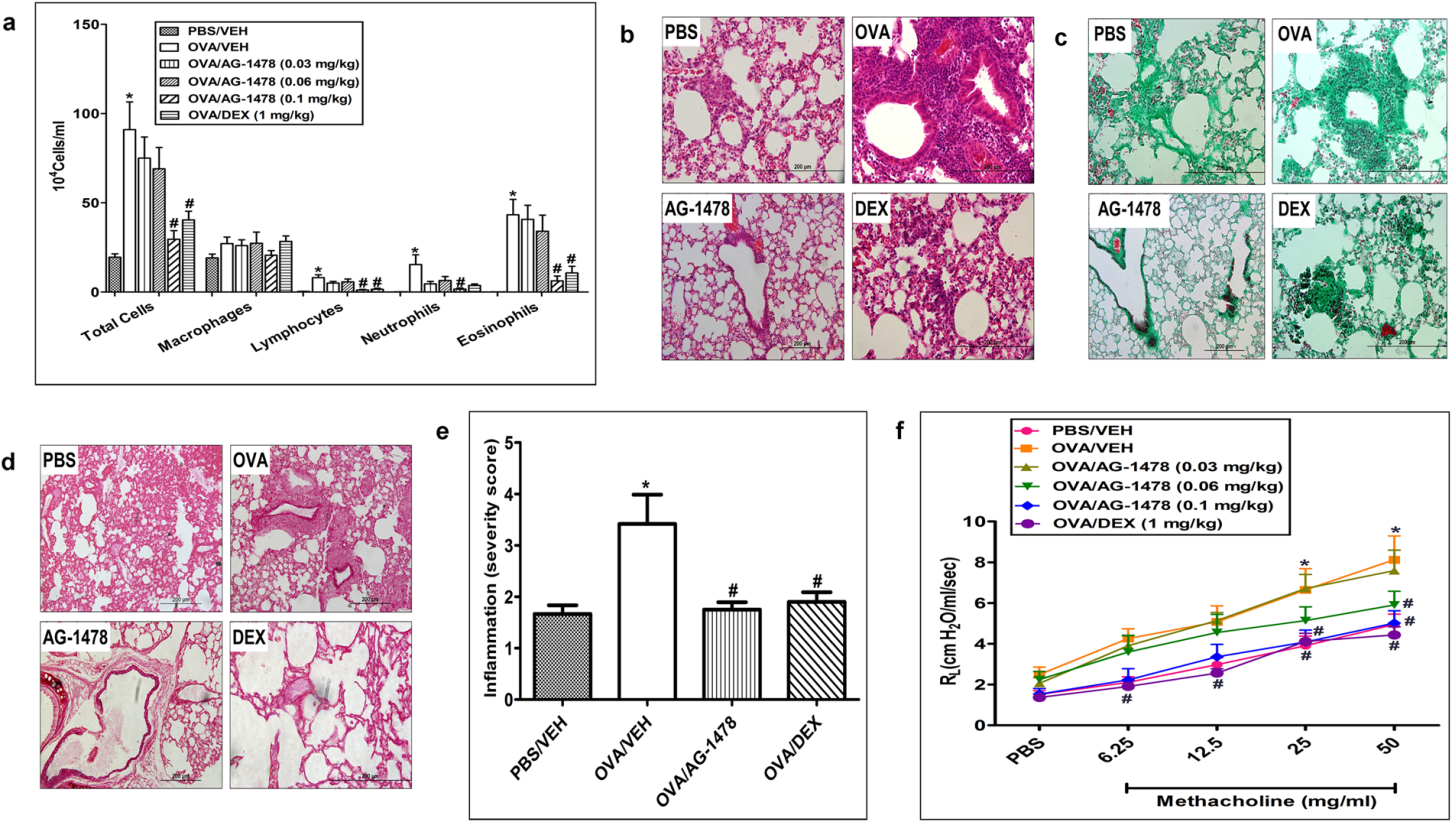
Effect of AG-1478 on total and differential cells, histology and airway hyperresponsiveness. Effect of AG-1478 (0.03, 0.06 and 0.1 mg/kg; *i.p*.) on OVA-induced change in total BALF cell count, eosinophils, lymphocytes, neutrophils and macrophage **(a)**. Treatment with AG-1478 dose dependently inhibited the OVA-induced increase in total cell influx, eosinophils, lymphocytes and neutrophils in the airways (*P* < 0.05). Data are expressed as mean ± SEM (*n* = 11–18). *P < 0.05 *versus* time-matched PBS-challenged mice. ^#^ *P* < 0.05 *versus* time-matched OVA-challenged mice. Representative low-magnification light photomicrographs display H&E staining **(b)**, Masson’s Trichrome staining **(c)** and PAS stain **(d)** of whole lung samples from PBS-challenged and vehicle treated (PBS) (*i.p*. treated; *n* = 6), OVA-challenged and vehicle treated **(OVA)** (i.p. treated; *n* = 6), OVA-challenged and AG-1478 treated (AG-1478) (0.1 mg/kg; *i.p*. treated; *n* = 6) and OVA-challenged and dexamethasone treated (DEX) (1 mg/kg; *i.p*. treated; *n* = 6) groups. OVA-challenged/vehicle-treated mice showed marked peribronchial and perivascular inflammatory cell infiltrations compared with PBS-challenged mice. Treatment with AG-1478 (0.1 mg/kg; i.p. treated) resulted in significant (*P* < 0.05) reduction in the peribronchial and perivascular dark-staining inflammatory cell infiltration **(b)**, peribronchial and perivascular fibrosis **(c)** and bronchial mucus production and goblet cell hyper/metaplasia **(d)** compared with the OVA-challenged mice and was comparable to dexamethasone treated group. Effect of AG-1478 (0.1 mg/kg) on inflammation severity score is shown in **(e)**. Data are expressed as mean ± SEM (*n* = 6). *P < 0.05 *versus* time-matched PBS-challenged mice. ^#^ *P* < 0.05 *versus* time-matched OVA-challenged mice. Effect of AG-1478 (0.03 mg/kg, 0.06 mg/kg and 0.1 mg/kg) and dexamethasone (1 mg/kg) on OVA-induced AHR to inhaled methacholine **(f)**. Airway responsiveness measurements were done 24 hs after the last challenge. OVA challenged mice had significant (P < 0.05) AHR compared with the PBS/Veh group and this was reduced following treatment with AG-1478 (0.1 mg/kg). Data are expressed as mean ± SEM (*n* = 12–19). *P < 0.05 *versus* time-matched PBS-challenged mice. ^#^ *P* < 0.05 *versus* time-matched OVA-challenged mice.

We next assessed the effects of AG1478 on OVA- induced inflammatory cell influx, airway remodeling and AHR. OVA challenge induced airway inflammation as shown by the significant (*P* < 0.05) increase in total cell influx (91 ± 15.6 *versus* 19.5 ± 1.9 (×10^4^) cells/ml BAL fluid, *P* < 0.05; n = 15); and differential cells, such as lymphocytes (8 ± 1.7 *versus* 0.3 ± 0.1 (×10^4^) cells/ml BAL fluid), neutrophils (15.4 ± 5.5 *versus* 0.1 ± 0.1 (×10^4^) cells/ml BAL fluid) and eosinophils (43.3 ± 8.6 *versus* 0.1 ± 0.0 (×10^4^) cells/ml BAL fluid) (Fig. 2a). OVA challenge also induced significant (*P* < 0.05) airway remodeling as evidenced by the severe and marked perivascular and peribronchial inflammation (H&E stain), airway fibrosis (Masson’s Trichrome stain) and goblet cell hyper/metaplasia (PAS stain) as confirmed by the inflammation severity score (Fig. 2b–e). Furthermore, treatment with AG1478 (0.03 mg/kg, 0.06 mg/kg and 0.1 mg/kg) resulted in a significant (*P* < 0.05) dose-dependent decrease in the inflammatory cell influx (Fig. 2a) and at the high dose of 0.1 mg/kg resulted in almost complete amelioration of the histopathological airway remodeling (Fig. 2b–e).

The OVA-induced inflammation and airway modeling resulted in AHR (Fig. 2f) as evidenced by the increase in lung resistance (R_L_) to methacholine and was significantly (P < 0.05) different at doses 25 and 50 mg/ml (6.6 ± 1.0 and 8.1 ± 1.2 *versus* 3.9 ± 0.5 and 4.9 ± 0.5 cm H_2_O/ml per second, respectively, compared to the PBS control) (Fig. 2f). Treatment with AG1478 dose-dependently reduced the OVA induced-AHR, and at the highest dose (0.1 mg/kg) resulted in a significantly (P < 0.05) lower average R_L_ at doses 25 and 50 mg/ml (4.1 ± 0.6 and 5.0 ± 0.6 *versus* 6.6 ± 1.0 and 8.1 ± 1.2 cm H_2_O/ml per second) and was comparable to the effects of dexamethasone (1 mg/kg) treated group (Fig. 2f).

### Effect of EGF on mononuclear, neutrophil, and eosinophil chemotaxis *in vitro*

To further understand the mechanism(s) by which EGF/EGFR is involved in inducing inflammatory cell influx in the asthma phenotype, we examined the direct and indirect effects of EGF/EGFR signaling on mononuclear and neutrophil chemotaxis *in vitro*. In the first set of experiments, we studied the direct effects of EGF (0.001, 0.01, 0.1, 1, and 10 µg/ml) on spleen-derived mononuclear cells and bone-marrow derived neutrophil chemotaxis. EGF did not induce any direct chemotactic response on either of the cell types (data not shown).

**Figure 3:**
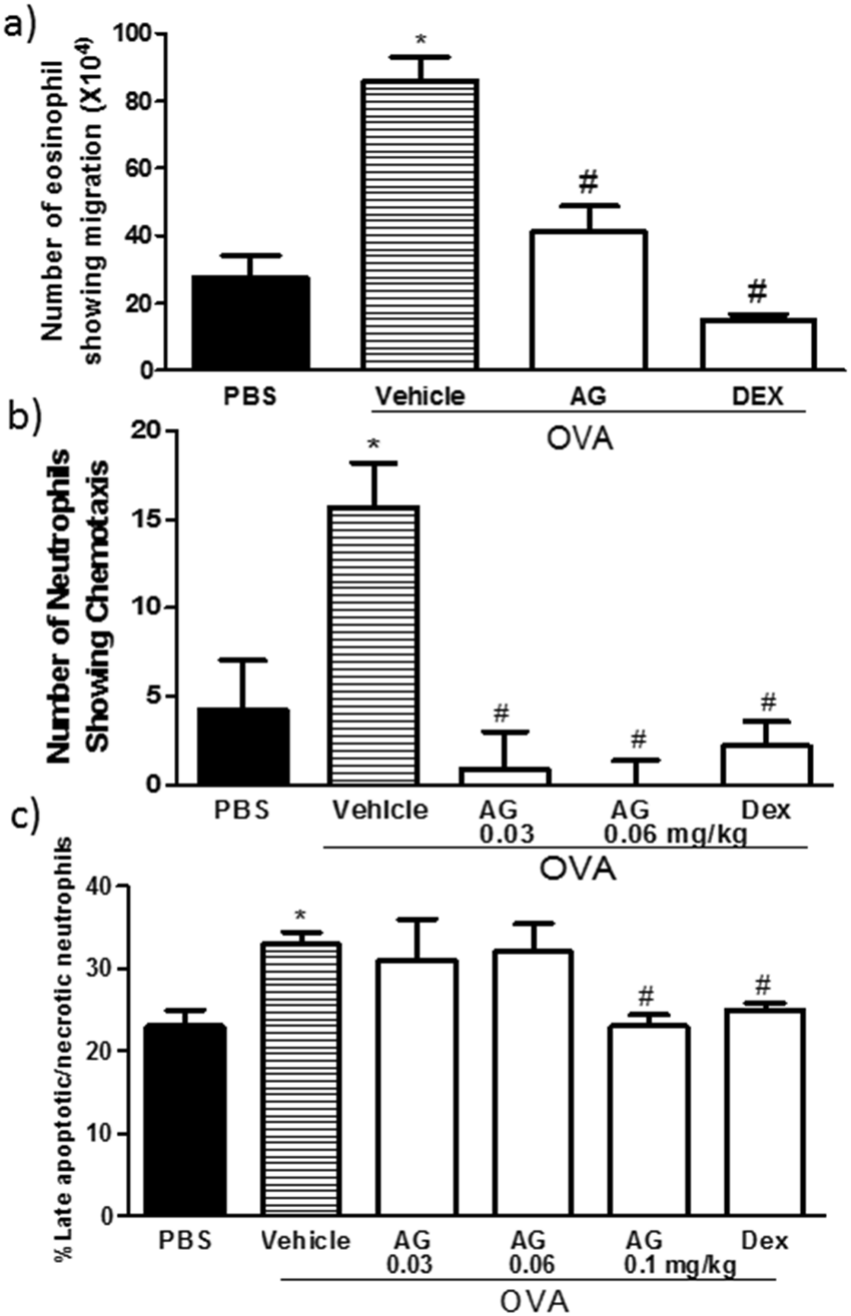
Effect of AG-1478 on neutrophil and eosinophil cell chemotaxis and apoptosis Effect of AG-1478 on eosinophil **(a)** neutrophil **(b)** chemotaxis, and neutrophil apoptosis **(c)**. Data are expressed as mean ± SEM (*n* = 3–4). *P < 0.05 *versus* time-matched PBS-challenged mice. ^#^ *P* < 0.05 *versus* time-matched OVA-challenged mice.

Therefore, to confirm the involvement of EGFR in inflammatory cell chemotaxis *in vitro*, we then assessed whether BALF, derived from OVA-challenged mice, may act as a stimulant for eosinophil or neutrophil cell chemotaxis and whether if this would be inhibited by BALF from AG1478 treated mice. Indeed, BALF from OVA-challenged mice induced a significant increase in eosinophil and neutrophil chemotaxis compared with the PBS-challenged mice and BALF from AG1478 treated mice ameliorated the OVA-induced eosinophil and neutrophil chemotaxis (*P* < 0.05) to levels similar to those observed in the dexamethasone treated group (Fig. 3a,b).

### Effect of EGF on neutrophil apoptosis *in vitro*

In these studies, we also examined both the direct effects of EGF (0.02, 0.1, 0.2, 2 µg/ml) and that of BALF from OVA-challenged and OVA-challenged/AG1478-treated mice on neutrophil apoptosis. Whilst EGF did not have any direct effect on neutrophil apoptosis (data not shown), BALF from AG178-treated group significantly decreased the percentage of late apoptotic/necrotic neutrophils relative to the OVA-challenged mice (Fig. 3c) and was comparable to the dexamethasone (1 mg/kg) group. However, there was no change observed in the percentage of viable neutrophils (data not shown).

### Effect of AG1478 on downstream signaling pathways involving ERK1/2 and PI3Kδ/Akt and the transcriptional factor NFκB

**Figure 4:**
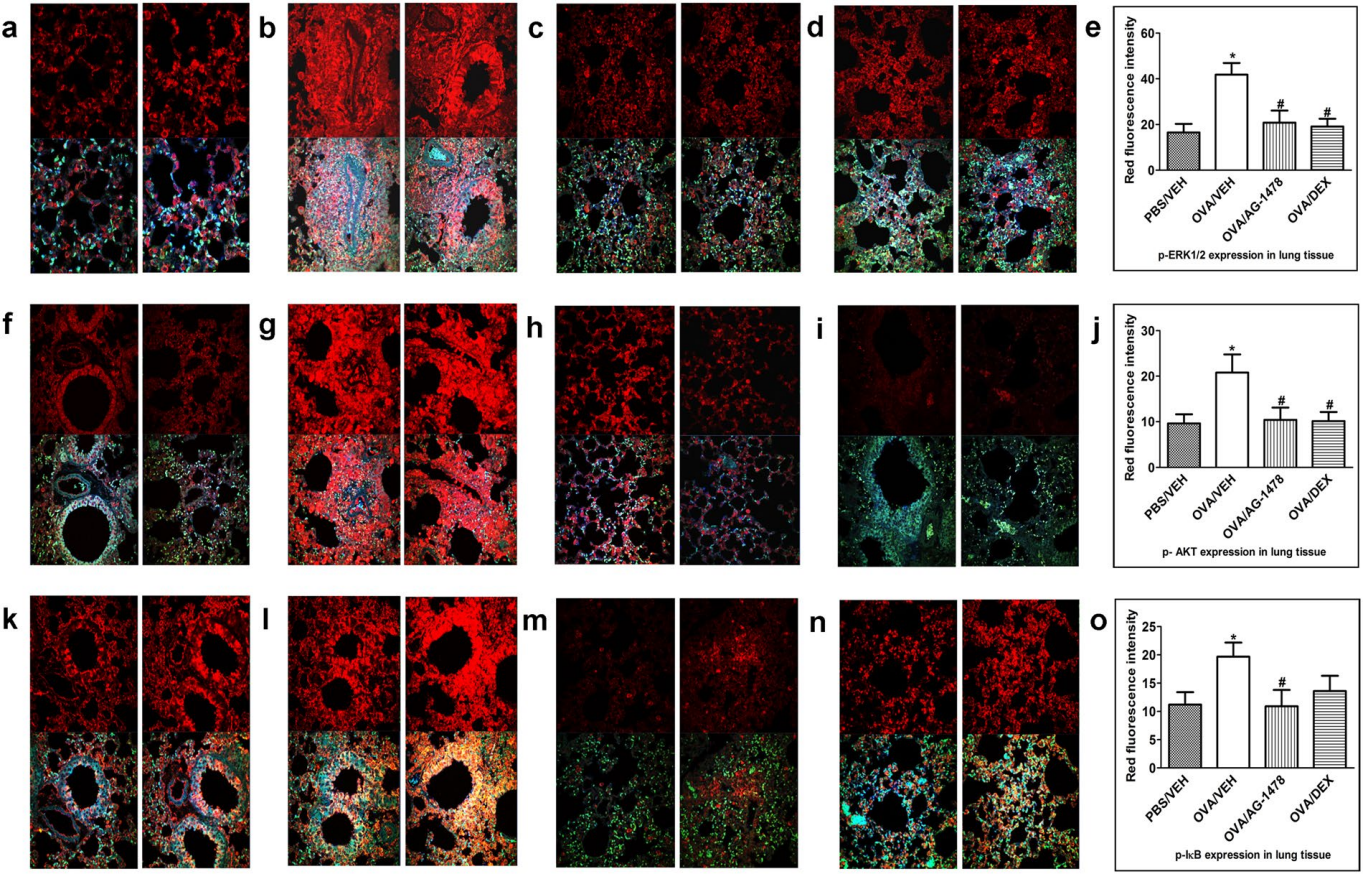
Effect of AG1478 on immunofluorescence detection of phosphorylated ERK1/2, Akt and IκB in lung sections (**a–o**). Lung sections were taken from different treatment groups - PBS/Veh **(a**,**f**,**k)**; OVA/Veh **(b,g,i)**; OVA/AG-1478 (0.1 mg/kg) **(c,h,m)** and OVA/Dex (1 mg/kg) (**d**,**i**,**n**) and were immunostained against phosphorylated ERK1/2 **(a**–**d)**, phosphorylated Akt **(f**–**i**) and phosphorylated IKB **(k**–**n**). Treatment with AG-1478 (0.1 mg/kg) significantly (P < 0.05) lowered the phospho levels of ERK1/2, Akt and IκB **(c** and **e**,**h** and **j**,**m** and **o)**. Immunofluorescence (Alexa Fluor) signals are shown on the left side of panels are overlaid with DAPI stain on the right side to show tissue architecture for the conditions indicated. Quantitative assessment of fluorescence intensity of phospho-ERK1/2 (**e**), phospho-Akt (**j**) and phospho-IκB (**o**). (arbitrary units). Data are expressed as mean ± SEM (*n* = 3). *P < 0.05 *versus* time-matched PBS-challenged mice. ^#^ *P* < 0.05 *versus* time-matched OVA-challenged mice.

To establish whether ERK1/2 and PI3Kδ/Akt dependent signaling pathways, and the transcriptional factor NFκB, are downstream of EGFR activation, we assessed them effect of inhibiting EGFR on their phosphorylation. OVA challenge induced a significant (*P* < 0.05) increase in the phosphorylation of ERK1/2 (Fig. 4b and e), Akt (Fig. 4g and j) and IκB (a surrogate marker for NFκB activation) (Fig. 4l and o) compared to the PBS-control group (Fig. 4a,e,f,j,k and o). Treatment with AG-1478 (0.1 mg/kg) significantly (*P* < 0.05) reduced the OVA-induced increased phosphorylation of ERK1/2 (Fig. 4c and e), Akt (Fig. 4h and j) and IκB (Fig. 4m and o) to levels similar to the dexamethasone (1 mg/kg) treated group (Fig. 4d,e,i,j,n and o).

### Effect of SU6656 on EGFR and downstream ERK1/2 in the OVA- induced asthma phenotype

**Figure 5:**
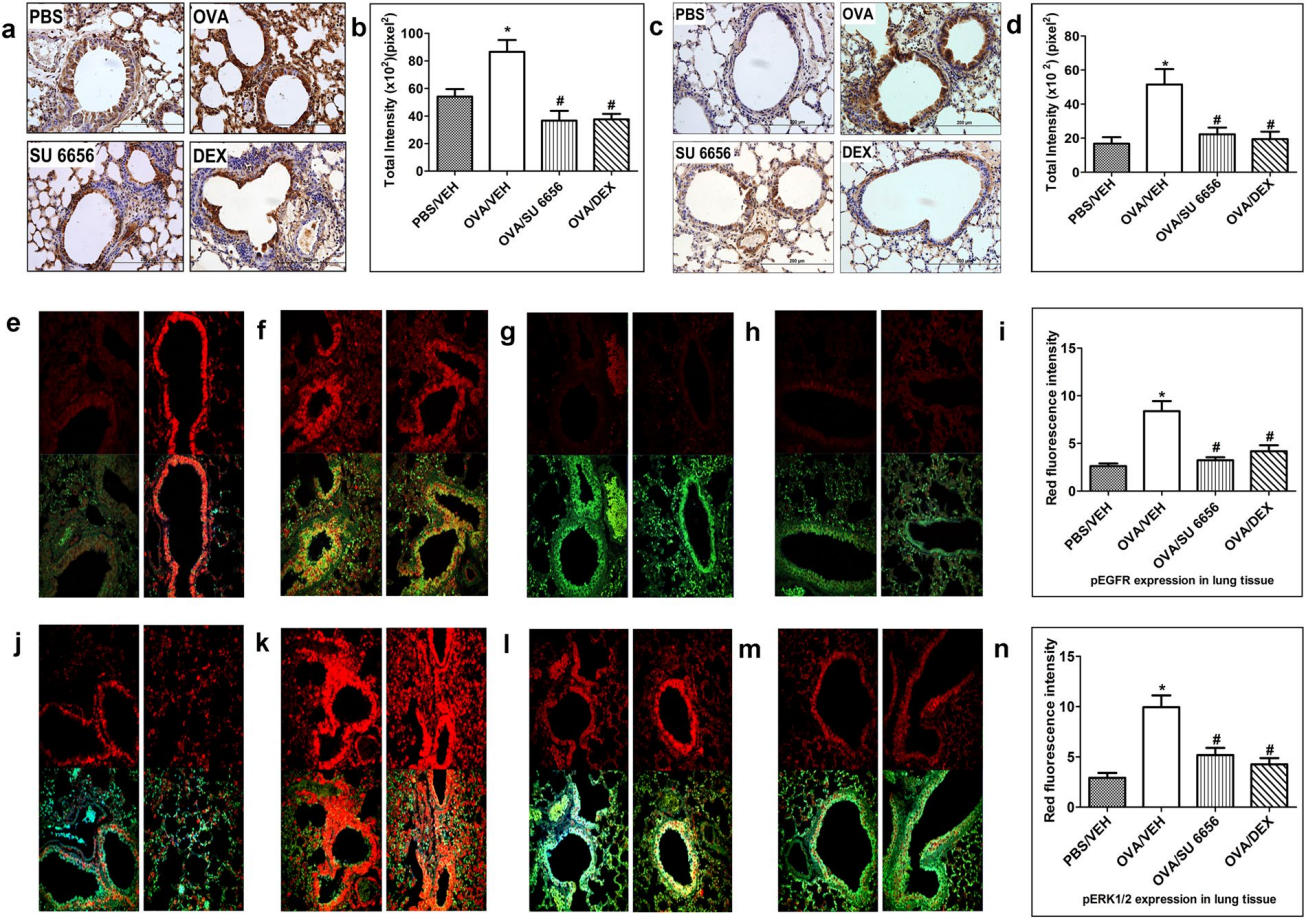
Effect of SU6656 on Immunohistochemistry and Immunofluorescence. Total EGFR immunohistochemical staining **(a)** and phosphorylated EGFR immunohistochemical staining **(c)** distribution photomicrographs of sections of the lung from PBS-challenged and vehicle treated (PBS) (i.n. treated; *n* = 3), OVA-challenged and vehicle treated (OVA) (i.n. treated; *n* = 3), OVA-challenged and SU6656 treated (SU6656) (8 mg/kg; i.n. treated; *n* = 3), OVA-challenged and dexamethasone treated (DEX) (1 mg/kg; i.n. treated; *n* = 3) groups. Analysis of the total EGFR **(b)** and phosphorylated EGFR **(d)** immunoreactive area in the lung sections of the different treatment groups. Data are expressed as mean ± SEM (*n* = 3). *P < 0.05 *versus* time-matched PBS-challenged mice. ^#^ *P* < 0.05 *versus* time-matched OVA-challenged mice. Immunofluorescence detection of p-EGFR **(e–i)** and p- ERK1/2 **(j**–**n)** in lung sections. Lung sections were taken from different treatment groups PBS/Veh **(e** and **j)**; OVA/Veh **(f** and **k)**; SU6656 (8 mg/kg) **(g** and **i)** and OVA/Dex (1 mg/kg) **(h** and **m)** and were immunostained against phosphorylated EGFR **(e**–**h**) and phosphorylated ERK1/2 **(j**–**m**). Immunofluorescent (Alexa Fluor) signals are shown on the left side of panels are overlaid with DAPI stain on the right side to show tissue architecture for the conditions indicated. Quantitative assessment of fluorescence intensity of phosphor-EGFR **(i)** and phosphor-ERK1/2 **(n)** (arbitrary units). Data are expressed as mean ± SEM (*n* = 3). *P < 0.05 *versus* time-matched PBS-challenged mice. ^#^ *P* < 0.05 *versus* time-matched OVA-challenged mice.

**Figure 6:**
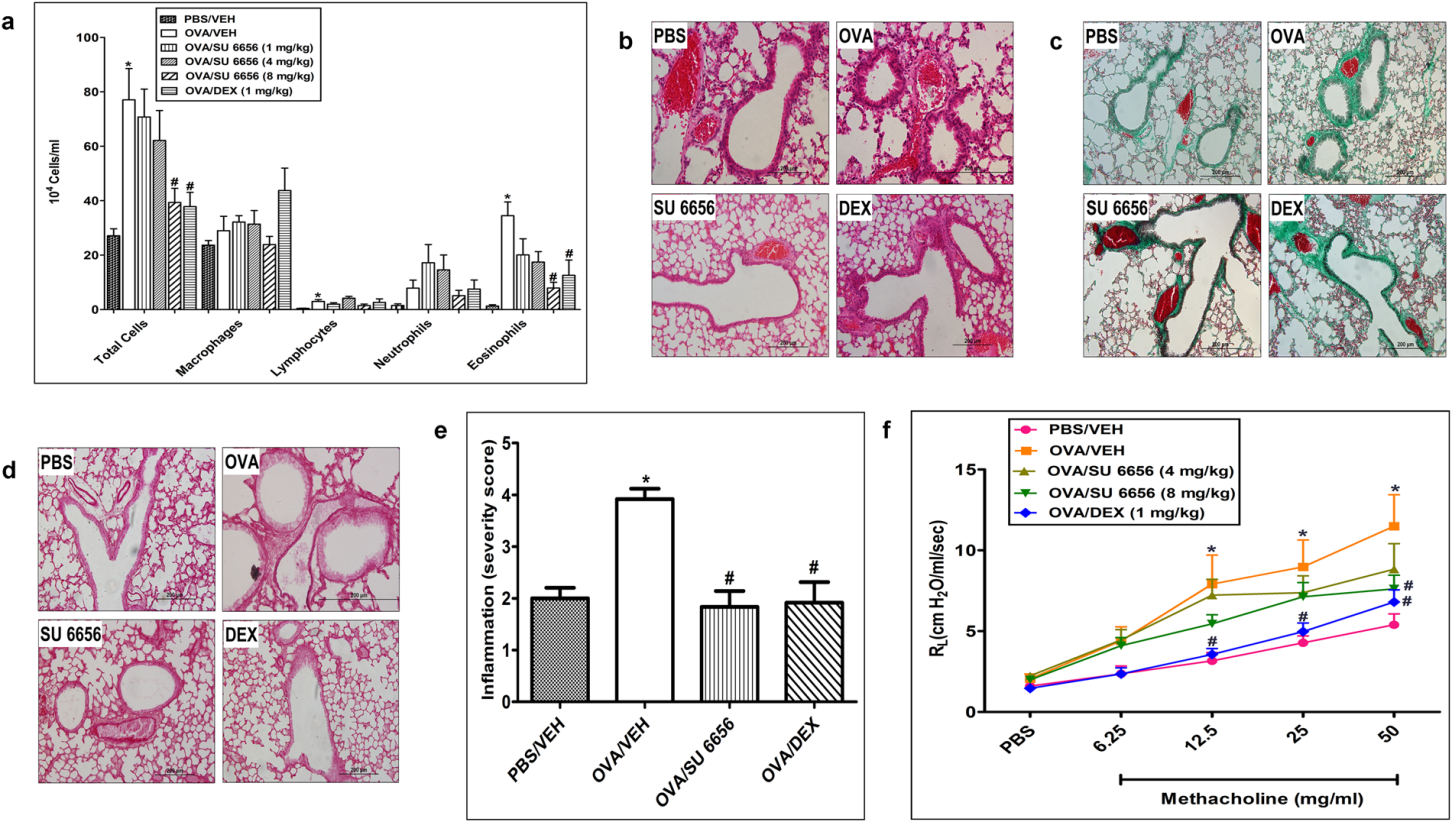
Effect of SU6656 on total and differential cells, histology and airway hyperresponsiveness. Effect of SU6656 (1, 4 and 8 mg/kg; i.n.) on OVA-induced change in total BALF cell count, eosinophils, lymphocytes, neutrophils and macrophage **(a)**. Treatment with SU6656 dose dependently inhibited the OVA-induced increase in total cell influx, eosinophils, lymphocytes and neutrophils in the airways. Data are expressed as mean ± SEM (*n* = 10–26). *P < 0.05 *versus* time-matched PBS-challenged mice. ^#^ *P* < 0.05 *versus* time-matched OVA-challenged mice. Representative low-magnification light photomicrographs display H&E staining **(b)**, Masson’s Trichrome staining **(c)** and PAS stain **(d)** of whole lung samples from PBS-challenged and vehicle treated (PBS) (i.n. treated; *n* = 6), OVA-challenged and vehicle treated **(OVA)** (i.n. treated; *n* = 6), OVA-challenged and SU6656 treated (SU6656) (8 mg/kg; i.n. treated; *n* = 6), OVA-challenged and dexamethasone treated (DEX) (1 mg/kg; i.n. treated; *n* = 6) groups. Treatment with SU6656 (8 mg/kg; *i.n*. treated) resulted in significant (P < 0.05) reduction in the peribronchial and perivascular inflammatory cell infiltration **(b)**, peribronchial and perivascular fibrosis **(c)** and bronchial mucus production and goblet cell hyper/metaplasia (**d)** compared with the OVA-challenged mice and was comparable to dexamethasone treated group. Effect of SU6656 (8 mg/kg) on inflammation severity score is shown in **(e)**. Data are expressed as mean ± SEM (*n* = 6). *P < 0.05 *versus* time-matched PBS-challenged mice. ^#^ *P* < 0.05 *versus* time-matched OVA-challenged mice. Effect of SU6656 (4 mg/kg and 8 mg/kg) and dexamethasone (1 mg/kg) on OVA-induced AHR to inhaled methacholine **(f)**. Treatment with 4 mg/kg and 8 mg/kg dose of SU6656 reduced the AHR compared with the OVA challenged group. Data are expressed as mean ± SEM (*n* = 7–12).

To investigate the role of Src in the transactivation of EGFR and subsequent activation of the ERK1/2 signaling pathway, we administered SU6656 as a non-selective inhibitor of SFK. Treatment with SU6656 (8 mg/kg) resulted in a significant (P < 0.05) decrease in both the expression (Fig. 5a and b) and the phosphorylation of EGFR (Fig. 5c,d,g and i). Furthermore, Src inhibition also significantly reduced the phosphorylated levels of ERK1/2 (Fig. 5l and n). The inhibitory effects of SU6656 on EGFR and ERK1/2 phosphorylation were similar to the effects of dexamethasone (Fig. 5a,b,c,d,h,i,m and n). Treatment with SU6656 (1, 4 and 8 mg/kg) also resulted in a significant (*P* < 0.05) dose-dependent decrease in the inflammatory cell influx (Fig. 6a) with the maximal dose achieving a near-complete amelioration of the histopathological airway remodeling compared to control group and the effects were comparable to the dexamethasone treated group (Fig. 6b–e). Likewise, SU6656 at doses 4 and 8 mg/kg dose-dependently reduced the AHR and the maximal dose (8 mg/kg) significantly (*P* < 0.05) lowered average R_L_ at 50 mg/ml dose of methacholine (7.6 ± 0.8 *versus* 11.5 ± 1.9 cm H_2_O/ml/sec) in comparison with the OVA-challenged/vehicle-treated group, and was comparable to the effects of dexamethasone (1 mg/kg) treated group (Fig. 6f).

### Effect of PD 89059, IC-87114 and BAY 11-7085on OVA-induced inflammatory cell influx, airway remodeling and AHR

To establish the role and contribution of the ERK1/2 and PI3Kδ/Akt dependent - pathways and NFκB in the OVA-induced inflammatory cell influx, airway remodeling and AHR, we used their inhibitors PD 98059, IC-87114 and BAY 11-7085, respectively.

**Figure 7:**
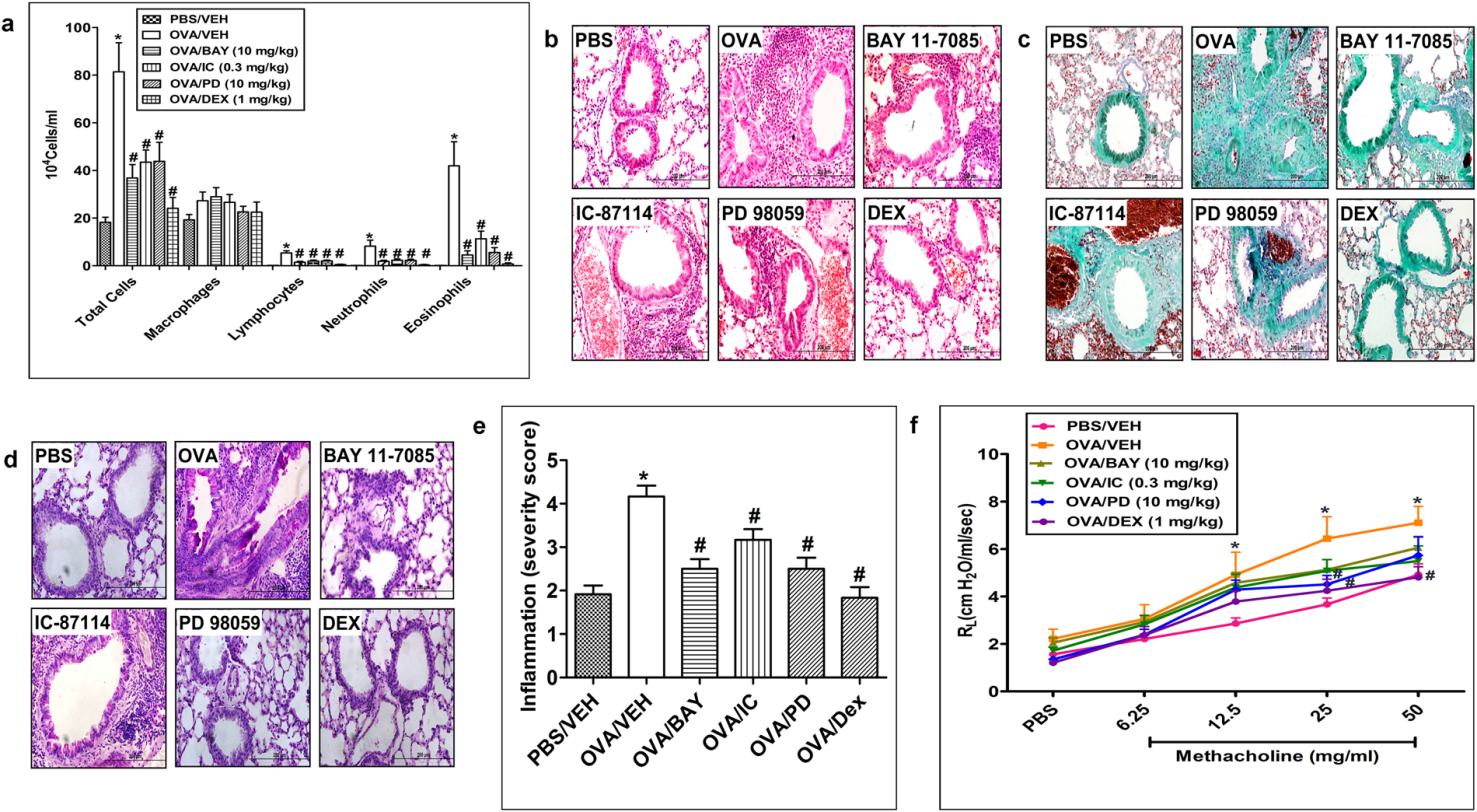
Effect of PD 89059, IC-87114 and BAY 11-7085 on total and differential cells, histology and airway hyperresponsiveness. Effect of PD 98059, IC-87114 and BAY 11-7085 and on OVA-induced change in total BALF cell count, eosinophils, lymphocytes, neutrophils and macrophages **(a)**. Treatment with Effect of PD 98059, IC-87114 and BAY 11-7085 inhibited the OVA-induced increase in total cell influx, eosinophils, lymphocytes and neutrophils in the airways. Data are expressed as mean ± SEM (*n* = 10–30). *P < 0.05 *versus* time-matched PBS-challenged mice. ^#^ *P* < 0.05 *versus* time-matched OVA-challenged mice. Representative low-magnification light photomicrographs display H&E staining **(b)**, Masson’s Trichrome staining **(c)** and PAS stain **(d)** of whole lung samples from PBS vehicle (*n* = 6), OVA-challenged (vehicle treated; i.p. *n* = 6), OVA-challenged/ PD 98059 (10 mg/kg; i.p. treated; *n* = 6) treated OVA-challenged/IC-87114 (0.3 mg/kg; i.p. treated; *n* = 6), OVA-challenged/BAY 11-7085 (10 mg/kg; i.p. treated; *n* = 6) and OVA-challenged/dexamethasone (1 mg/kg; i.p. treated; *n* = 6) treated groups. Treatment with PD 98059, IC-87114 and BAY 11-7085 (i.p. treated; *n* = 6) resulted in resulted in significant (P < 0.05) reduction in the peribronchial and perivascular dark-staining inflammatory cell infiltration **(b)**, peribronchial and perivascular fibrosis **(c)** and bronchial mucus production and goblet cell hyper/metaplasia **(d)** compared with the OVA-challenged mice and was comparable to dexamethasone treated group. Effect of BAY 11-7085 (10 mg/kg), IC-87114 (0.3 mg/kg) and PD 98059 (10 mg/kg) on inflammation severity score is shown in **(e)**. Data are expressed as mean ± SEM (*n* = 6). *P < 0.05 *versus* time-matched PBS-challenged mice. ^#^ *P* < 0.05 *versus* time-matched OVA-challenged mice. Effect of PD 98059 (10 mg/kg), IC-87114 (0.3 mg/kg) and BAY 11-7085 (10 mg/kg) on OVA-induced AHR to inhaled methacholine **(f)**. Treatment with PD 98059 (10 mg/kg), IC-87114 (0.3 mg/kg) and BAY 11-7085 (10 mg/kg) did not significantly (P > 0.05) reduce the OVA induced AHR. Data are expressed as mean ± SEM (*n* = 7–13). *P < 0.05 *versus* time-matched PBS-challenged mice. ^#^ *P* < 0.05 *versus* time-matched OVA-challenged mice.

Treatment with PD 89059 (10 mg/kg), IC-87114 (0.3 mg/kg) and BAY 11-7085 (10 mg/kg), significantly (*P* < 0.05) reduced the OVA- induced inflammatory cell influx into the airways (Fig. 7a) and the histopathological airway remodeling (Fig. 7b–e). However, these treatments did not significantly improve OVA induced-AHR (*P* > 0.05). Of note, the observed reduction in the histopathological airway remodeling induced by PD 89059, IC-87114 and BAY 11-7085 were less effective as compared to the reduction seen with AG 1478 and SU6656.

## Discussion

**Figure 8:**
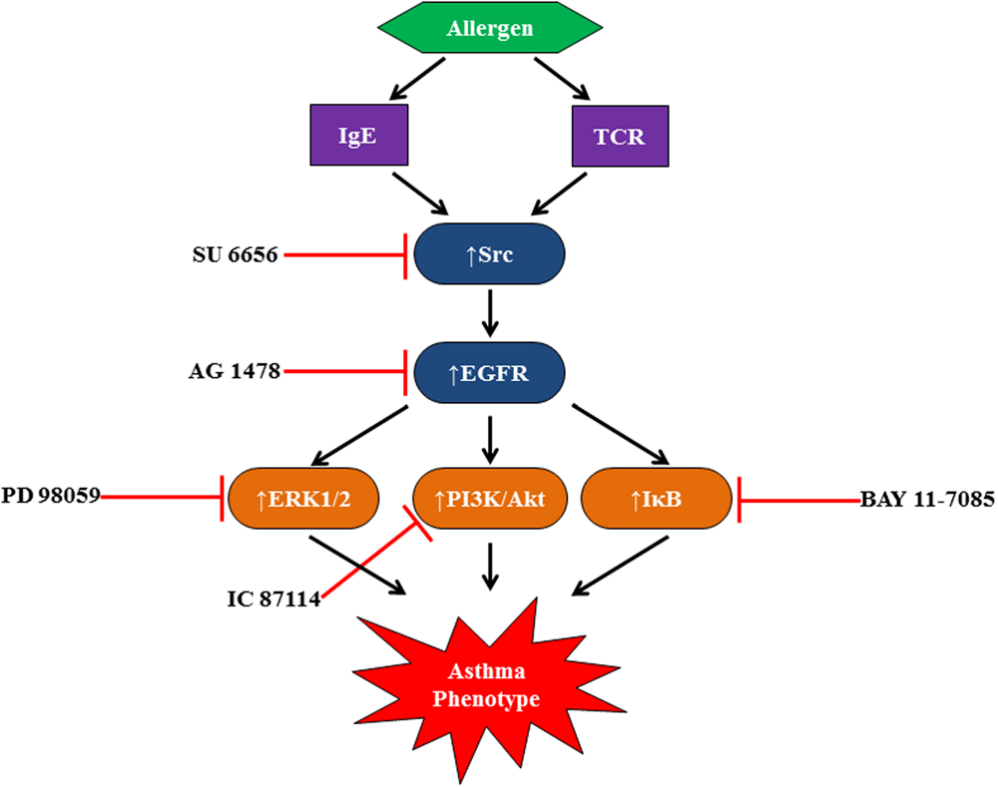
A diagrammatic summary of the Src/EGFR/ ERK1/2, PI3Kδ/Akt and NFκB signal transduction pathways hypothesized to be involved in asthma. Exposure to allergen in the airways activates receptors such as T-cell receptor and IgE, expressed on numerous cell types to induce allergic immune responses. Activation of these immune cell receptors then activates SFK, which in turn activates EGFR. EGFR signaling via ERK1/2, PI3K/Akt, and NFκB effector molecules regulates the characteristic features of asthma pathogenesis.

In a murine asthma model, we show that SFK plays a critical role in the transactivation of EGFR and the subsequent activation of multiple downstream signaling pathways involving ERK1/2 and PI3Kδ/Akt and the transcriptional factor NFκB, which ultimately lead to the development of the allergic airway inflammatory response (see Fig. 8).

Our data showed that increased EGFR mRNA and protein expression as well as phosphorylation of EGFR resulted in several of the pathophysiological features of asthma. These include increased inflammatory cell influx, airway remodeling such as perivascular and peribronchial inflammation, airway fibrosis, goblet cell hyper/metaplasia and AHR. In terms of pulmonary inflammatory cell influx, we observed that EGFR inhibition, with a specific EGFR tyrosine kinase inhibitor, AG1478, significantly reduced the OVA-induced eosinophil and neutrophil chemotaxis. Interestingly, exposure of naïve neutrophils to EGF did not induce cell migration, consistent with previous observations in human neutrophils^37^. A potential explanation for these observations are that neutrophils from naïve and not from mice with OVA-induced asthma were used and/or that EGF is a not a direct neutrophil chemotactic agent or indeed that other EGFR receptor ligands may be more important in this regard. However, BALF from OVA challenged mice induced significant eosinophil and neutrophil chemotaxis which was blocked by EGFR inhibition implying that EGFR regulates chemotaxis of two important inflammatory cells in asthma pathogenesis by indirect mechanisms.

In addition, our data shows that OVA induced significant neutrophil cell death through late apoptosis/necrosis. This effect was attenuated by EGFR inhibition, to the same levels observed with dexamethasone treatment, suggesting that EGFR signaling is important in promoting late neutrophil apoptosis/necrosis. These findings are inconsistent with published studies in human asthma demonstrating that EGF stimulation of the asthmatic epithelium induces pro-survival factors that can inhibit neutrophil spontaneous apoptosis^14^ and that defective neutrophil apoptosis has been reported in severe allergic asthma^38^

In our asthma model, enhanced EGFR signaling was also associated with increased signaling via, ERK1/2, PI3Kδ/Akt and the transcriptional factor NFκB. The fact that these allergic airway inflammatory responses and more importantly the EGFR-triggered signaling perturbations were significantly attenuated by AG1478, confirms the critical role played by EGFR in mediating the pathophysiology of asthma. At the downstream level, it supports the regulatory role of ERK1/2, PI3Kδ/Akt and NFκB as effectors of EGFR transduction. More importantly, we show that inhibition of the SFK, with SU6656, inhibited EGFR phosphorylation and its associated downstream signaling which suggests that SFK is an important mediator of EGFR transactivation in this model of asthma. As SU6656 is a non-selective inhibitor of SFK, the exact Src kinase/s involved in EGFR transactivation is/are yet to be determined.

In addition to the inhibition of Src and EGFR leading to the normalization of perturbed allergic airway inflammatory responses, we further showed that the selective inhibition of the downstream effects of either ERK1/2 by PD 98059, PI3Kδ by IC-87114 or NFκB by BAY 11-7085 also reversed OVA-induced allergic airway inflammation. However, this was to a lesser extent compared to AG1478 or SU6656 in that the overall severity score with the pathway selective inhibitor was higher than that noted with the AG1478 and SU6656. Moreover, treatment with PD 98059, IC-87114 and BAY 11-7085 did not significantly inhibit AHR. Therefore, upstream inhibition, at the level of Src or EGFR, appears to be more effective than blockade of the individual downstream pathways involving ERK1/2, PI3Kδ/Akt or the transcriptional factor NFκB – a finding that may have important clinical implications in the treatment of asthma.

Emerging evidence suggests an important role for EGFR in human asthma pathophysiology^11, 14^. Recent studies, utilizing similar animal models of asthma, using different EGFR tyrosine kinase inhibitors, such gefitnib and erlotinibin showed marked reductions in EGFR phosphorylation accompanied by dampening of several features of asthma, such cellular influx and airway remodeling^5, 39^. Of clinical relevance, a recent clinical study conducted using *ex vivo* lung tissue from patients with COPD, and showed that the EGFR inhibitor BIBW 2948 had some efficacy in inhibiting EGFR phosphorylation and a tendency toward reducing mucous metaplasia thus establishing a “proof of concept” for beneficial effects of EGFR inhibition in the treatment of chronic airway inflammatory diseases^40^. However, due to the high side effect profile^41^ which may be drug specific and/or target receptor specific, as well as the potential for drug resistance^42, 43^, as is common in patients receiving EGFR inhibitors, alternative strategies to inhibit the EGFR signaling may be more desirable.

Whilst there is evidence, that both EGF and EGFR are up-regulated in the asthmatic epithelium that may contribute to asthma pathophysiology^4, 44, 45^, experimental models of asthma have not consistently reported the upregulation of EGF ligand levels^13^. This perhaps suggests that other ligands such as HB-EGF, betacellulin (BTC), amphiregulin and/or ligand-independent EGFR transactivation such as via ROS, GPCR and SFKs may be more important^8, 46–49^. Indeed, we and others have recently shown that SFK mediate EGFR transactivation in a variety of disease states including cardiovascular diseases and cancer^20, 50^. Thus, these studies highlight an important role for SFK in upstream EGFR signaling in pathological states. In fact, SFK were the first of the non-receptor tyrosine kinases to be characterized and has several members such as the Fyn, Lyn, Yes, Hck^51, 52^ and play roles in both T cell receptor (TCR) and the high affinity receptor for IgE (FcεRI) signaling^51, 52^. The interplay between SFK and EGFR and the exact context of the Src/EGFR signaling pathway in the pathogenesis of asthma is poorly understood. However, it appears that critical upstream events initiated via the allergen/ TCR signaling pathway and the allergen-IgE/ FceRI receptor can activate SFK^51, 52^. Our finding that Src is an important transactivator of EGFR in asthma, therefore leads us to speculate that SFK may be a signaling hub and relay transducing pathways that could lead to airways inflammation, remodeling and AHR. As to which member of the SFK is more important is unclear as they are known to play in important role in driving the pathogenic responses in asthma^53–56^. Since our study used SU6656, a selective inhibitor of SFK, the exact member(s) of SFK involved in the transactivation of EGFR in the present study is unknown and requires further investigation.

We further showed that ERK1/2, PI3Kδ/Akt, NFκB are downstream effectors of Src/EGFR signaling in the driving the allergic airway inflammatory responses. Furthermore, selective inhibition of each of these downstream molecules resulted in improvement of many of the pathological features of asthma however, the overall improvement in the inflammation severity score for all the downstream effector molecules appeared to be less marked compared with the inhibition of the upstream EGFR or Src. More importantly, there was no significant improvement in the OVA-induced AHR following specific inhibition of PI3Kδ, ERK1/2 or NFκB in comparison with the inhibition of EGFR or SFK. These results imply that, whilst multiple downstream pathways of Src/EGFR contribute to the asthma phenotype, upstream inhibtion of Src/EGFR is more effective than selective inhibition of downstream pathways or transcriptional factors.

In conclusion, the data presented in this study show that, in a murine asthma model, Src-kinase mediates EGFR transactivation which in turn stimulates multiple downstream signaling pathways involving ERK1/2 and PI3Kδ and transcriptional factor NFκB to regulate allergic lung inflammation (Fig. 8). Further, blockade of any one of these downstream effectors is less effective than inhibition of the multiple pathways as attained with inhibition of either SFK or EGFR. Thus, whilst potential inhibition of the PI3Kδ, ERK1/2 and NFκB effector molecules may be of some benefit as a therapeutic strategy asthma, it is more likely that a greater therapeutic margin would be gained by blocking upstream targets such SFK or EGFR.

## References

[1] Holgate ST (2013). Mechanisms of asthma and implications for its prevention and treatment: a personal journey. Allergy Asthma Immunol Res.

[2] Carsin A (2016). Bronchial epithelium in children: a key player in asthma. Eur Respir Rev.

[3] Amishima M (1998). Expression of epidermal growth factor and epidermal growth factor receptor immunoreactivity in the asthmatic human airway. Am J Respir Crit Care Med.

[4] Puddicombe SM (2000). Involvement of the epidermal growth factor receptor in epithelial repair in asthma. FASEB journal: official publication of the Federation of American Societies for Experimental Biology.

[5] Song L (2016). The Chronic and Short-Term Effects of Gefinitib on Airway Remodeling and Inflammation in a Mouse Model of Asthma. Cell Physiol Biochem.

[6] Tan WL (2016). Novel therapeutic targets on the horizon for lung cancer. Lancet Oncol.

[7] Harskamp LR, Gansevoort RT, van Goor H, Meijer E (2016). The epidermal growth factor receptor pathway in chronic kidney diseases. Nat Rev Nephrol.

[8] Akhtar S (2009). Role of epidermal growth factor receptor (EGFR) in corneal remodelling in diabetes. Acta ophthalmologica.

[9] Benter IF, Yousif MH, Griffiths SM, Benboubetra M, Akhtar S (2005). Epidermal growth factor receptor tyrosine kinase-mediated signalling contributes to diabetes-induced vascular dysfunction in the mesenteric bed. British journal of pharmacology.

[10] Akhtar S, Yousif MH, Chandrasekhar B, Benter IF (2012). Activation of EGFR/ERBB2 via pathways involving ERK1/2, P38 MAPK, AKT and FOXO enhances recovery of diabetic hearts from ischemia-reperfusion injury. PloS one.

[11] Holgate ST (2000). Epithelial damage and response. Clinical and experimental allergy: journal of the British Society for Allergy and Clinical Immunology.

[12] Vargaftig BB, Singer M (2003). Leukotrienes mediate part of Ova-induced lung effects in mice via EGFR. American journal of physiology. Lung cellular and molecular physiology.

[13] Tamaoka M (2008). The epidermal growth factor receptor mediates allergic airway remodelling in the rat. Eur Respir J.

[14] Uddin M (2013). EGF-induced bronchial epithelial cells drive neutrophil chemotactic and anti-apoptotic activity in asthma. PLoS One.

[15] Burkhardt AL, Brunswick M, Bolen JB, Mond JJ (1991). Anti-immunoglobulin stimulation of B lymphocytes activates src-related protein-tyrosine kinases. Proceedings of the National Academy of Sciences of the United States of America.

[16] Pazdrak K, Justement L, Alam R (1995). Mechanism of inhibition of eosinophil activation by transforming growth factor-beta. Inhibition of Lyn, MAP, Jak2 kinases and STAT1 nuclear factor. J Immunol.

[17] Corey S (1993). Granulocyte macrophage-colony stimulating factor stimulates both association and activation of phosphoinositide 3OH-kinase and src-related tyrosine kinase(s) in human myeloid derived cells. EMBO J.

[18] Linnekin D, DeBerry CS, Mou S (1997). Lyn associates with the juxtamembrane region of c-Kit and is activated by stem cell factor in hematopoietic cell lines and normal progenitor cells. The Journal of biological chemistry.

[19] Randhawa V, Bagler G (2012). Identification of SRC as a potent drug target for asthma, using an integrative approach of protein interactome analysis and in silico drug discovery. Omics: a journal of integrative biology.

[20] Akhtar S (2012). Angiotensin-(1-7) inhibits epidermal growth factor receptor transactivation via a Mas receptor-dependent pathway. British journal of pharmacology.

[21] El-Hashim AZ (2012). Angiotensin-(1-7) inhibits allergic inflammation, via the MAS1 receptor, through suppression of ERK1/2- and NF-kappaB-dependent pathways. British journal of pharmacology.

[22] Lee KS, Lee HK, Hayflick JS, Lee YC, Puri KD (2006). Inhibition of phosphoinositide 3-kinase delta attenuates allergic airway inflammation and hyperresponsiveness in murine asthma model. FASEB journal: official publication of the Federation of American Societies for Experimental Biology.

[23] Figini M (1996). Evidence that epithelium-derived relaxing factor released by bradykinin in the guinea pig trachea is nitric oxide. American journal of respiratory and critical care medicine.

[24] Liu W (2008). Cell-specific activation profile of extracellular signal-regulated kinase 1/2, Jun N-terminal kinase, and p38 mitogen-activated protein kinases in asthmatic airways. The Journal of allergy and clinical immunology.

[25] Kampe M (2012). PI3-kinase regulates eosinophil and neutrophil degranulation in patients with allergic rhinitis and allergic asthma irrespective of allergen challenge model. Inflammation.

[26] El-Hashim AZ (2011). Effect of inhibition of the ubiquitin-proteasome-system and IkappaB kinase on airway inflammation and hyperresponsiveness in a murine model of asthma. Int J Immunopathol Pharmacol.

[27] Moteki H, Kimura M, Ogihara M (2011). Activation of extracellular-signal regulated kinase by epidermal growth factor is potentiated by cAMP-elevating agents in primary cultures of adult rat hepatocytes. Biological & pharmaceutical bulletin.

[28] Khajah MA, Fateel MM, Ananthalakshmi KV, Luqmani YA (2016). Anti-Inflammatory Action of Angiotensin 1–7 in Experimental Colitis. PLoS One.

[29] Pfaffl MW (2001). A new mathematical model for relative quantification in real-time RT-PCR. Nucleic Acids Res.

[30] Ezeamuzie CI, El-Hashim AZ, Renno WM, Edafiogho IO (2014). Antiallergic and antiasthmatic effects of a novel enhydrazinone ester (CEE-1): inhibition of activation of both mast cells and eosinophils. The Journal of pharmacology and experimental therapeutics.

[31] Queto T (2010). Inducible nitric oxide synthase/CD95L-dependent suppression of pulmonary and bone marrow eosinophilia by diethylcarbamazine. American journal of respiratory and critical care medicine.

[32] Khajah M, Millen B, Cara DC, Waterhouse C, McCafferty DM (2011). Granulocyte-macrophage colony-stimulating factor (GM-CSF): a chemoattractive agent for murine leukocytes *in vivo*. J Leukoc Biol.

[33] Lieber JG (2004). The *in vitro* production and characterization of neutrophils from embryonic stem cells. Blood.

[34] Ezeamuzie CI, Philips E (1999). Adenosine A3 receptors on human eosinophils mediate inhibition of degranulation and superoxide anion release. Br J Pharmacol.

[35] Hansel TT (1991). An improved immunomagnetic procedure for the isolation of highly purified human blood eosinophils. J Immunol Methods.

[36] Gomez-Cambronero J, Horn J, Paul CC, Baumann MA (2003). Granulocyte-macrophage colony-stimulating factor is a chemoattractant cytokine for human neutrophils: involvement of the ribosomal p70 S6 kinase signaling pathway. J Immunol.

[37] Uddin M (2008). Enhancement of neutrophil function by the bronchial epithelium stimulated by epidermal growth factor. The European respiratory journal.

[38] Uddin M (2010). Prosurvival activity for airway neutrophils in severe asthma. Thorax.

[39] Le Cras TD (2011). Epithelial EGF receptor signaling mediates airway hyperreactivity and remodeling in a mouse model of chronic asthma. American journal of physiology. Lung cellular and molecular physiology.

[40] Woodruff PG (2010). Safety and efficacy of an inhaled epidermal growth factor receptor inhibitor (BIBW 2948 BS) in chronic obstructive pulmonary disease. American journal of respiratory and critical care medicine.

[41] Kozuki T (2016). Skin problems and EGFR-tyrosine kinase inhibitor. Jpn J Clin Oncol.

[42] Li X (2016). Shikonin inhibits gefitinib-resistant non-small cell lung cancer by inhibiting TrxR and activating the EGFR proteasomal degradation pathway. Pharmacological research: the official journal of the Italian Pharmacological Society.

[43] Kuwano M, Sonoda K, Murakami Y, Watari K, Ono M (2016). Overcoming drug resistance to receptor tyrosine kinase inhibitors: Learning from lung cancer. Pharmacology & therapeutics.

[44] Davies DE, Polosa R, Puddicombe SM, Richter A, Holgate ST (1999). The epidermal growth factor receptor and its ligand family: their potential role in repair and remodelling in asthma. Allergy.

[45] Polosa R (2002). Expression of c-erbB receptors and ligands in the bronchial epithelium of asthmatic subjects. The Journal of allergy and clinical immunology.

[46] Daub H, Weiss FU, Wallasch C, Ullrich A (1996). Role of transactivation of the EGF receptor in signalling by G-protein-coupled receptors. Nature.

[47] Bokemeyer D, Schmitz U, Kramer HJ (2000). Angiotensin II-induced growth of vascular smooth muscle cells requires an Src-dependent activation of the epidermal growth factor receptor. Kidney international.

[48] Akhtar S (2015). Transactivation of ErbB Family of Receptor Tyrosine Kinases Is Inhibited by Angiotensin-(1-7) via Its Mas Receptor. PLoS One.

[49] Dhaunsi, G.S., Alsaeid, M. & Akhtar, S. Phytanic acid attenuates Insulin-like Growth Factor-1 activity via Nitric oxide-mediated gamma-Secretase activation in Rat Aortic Smooth Muscle Cells: Possible Implications for Pathogenesis of Infantile Refsum Disease. *Pediatr Res* (2016). 10.1038/pr.2016.258 27886192

[50] Yap TA, Macklin-Doherty A, Popat S (2016). Continuing EGFR inhibition beyond progression in advanced non-small cell lung cancer. Eur J Cancer.

[51] Tundwal K, Alam R (2012). JAK and Src tyrosine kinase signaling in asthma. Front Biosci (Landmark Ed).

[52] Kopec A, Panaszek B, Fal AM (2006). Intracellular signaling pathways in IgE-dependent mast cell activation. Arch Immunol Ther Exp (Warsz).

[53] Ramis I (2015). A novel inhaled Syk inhibitor blocks mast cell degranulation and early asthmatic response. Pharmacological research.

[54] Norton SK (2008). IL-10 suppresses mast cell IgE receptor expression and signaling *in vitro* and *in vivo*. The Journal of Immunology.

[55] Adachi T, Stafford S, Sur S, Alam R (1999). A novel Lyn-binding peptide inhibitor blocks eosinophil differentiation, survival, and airway eosinophilic inflammation. Journal of immunology.

[56] Li G (2013). Lyn mitigates mouse airway remodeling by downregulating the TGF-β3 isoform in house dust mite models. The Journal of Immunology.

